# Regional noise source location based on the time delays between station pairs from ambient noise interferometry

**DOI:** 10.1038/s41598-024-60313-1

**Published:** 2024-05-04

**Authors:** Ziqiang Lü, Qian Liu, Qinghan Kong, Jingwen Sun, Zemin Liu

**Affiliations:** 1 College of Mining, Liaoning Technical University, Fuxin, 123000 China; 2 Institute of Geophysics, China Earthquake Administration, Beijing, 100081 China

**Keywords:** Geophysics, Seismology

## Abstract

Identifying the location of a potential noise source assists in understanding the characteristics of the seismic or volcanic activity and provides valuable information for hazard assessment. Unlike the conventional waveform-based techniques that rebuild the source energy into the possible source region, we apply a simplified method to determine the absolute location of the noise source based on the station-pair time-delays from ambient noise interferometry. Synthetic tests demonstrate the robustness of the method and the locating precision is mainly influenced by the signal-to-noise ratio of the synthetic waveforms, and the higher frequency bandwidth source signals are more likely to result in accurate detection of the source. An application at the Central Tien Shan indicates that our method is capable of locating the known virtual source from the empirical Green’s functions. Furthermore, assuming a surface wave velocity, the depth of the source can be generally recovered from ambient noise interferometry in a simplified 3-D homogeneous model. The new method sheds light on applications of ambient noise interferometry for locating potential sources, making it suitable for detecting time-dependent behavior.

## Introduction

Accurate regional source locations provide essential information on the source mechanism, volcanic zone monitoring, and seismic hazard mitigation^1–3^. A typical source location method based on traveltime inversion has been proposed by Geiger^4^. Since the 1990s, nonlinear methods have been applied to search for the source location in the regular or stochastic model space to minimize the misfit between the theoretical and observed traveltimes, such as the genetic algorithm^5^, and the Monte Carlo technique^6^. Significant improvements have been introduced to enhance the performance of the traveltime-based methods, including the relative location method^7^, double-difference relocation method^2,8^, and cluster-based relocation methods^9,10^. The traveltime-based location methods generally require phase-picking of the first arrival body wave, which brings high measuring error for the low signal-to-noise ratio seismic waveforms, leading to unreliable location results from insufficient spatial network coverage. Moreover, these methods are often phase-picking on individual seismograms, making little use of the cross-correlation information between stations.

Instead of using the conventional traveltime-based methods, the waveform-based source location methods do not rely on identifying or selecting phases and can detect and locate the source with a relatively low signal-to-noise ratio^11^. Waveform-based methods operate on the principle of focusing on the source location by using a migration or imaging operator to rebuild the source energy into specific grid points, such as back projection imaging, beamforming, and coherence scanning^1,7,12^. Recently, various studies integrated waveform-based methods with cross-correlation techniques derived from seismic interferometry^13–17^. The potential source location is determined by stacking and imaging techniques, followed by applying a detection and picking criterion to identify the optimal source location^11^. These methods have been applied to induce microseismic monitoring^18–20^, volcanic tremor^21,22^, and regional seismicity^3,23^. Most of these methods use the direct P wave with generally weak source energy in comparison with the surface wave or constrain the source location in 2-D planar space. Moreover, waveform stacking and time reverse imaging require expensive computational effort compared to the traveltime-based methods.

Ambient noise interferometry is a powerful technique to construct surface waves for imaging the structure of the Earth’s subsurface by utilizing cross-correlation from the noise signals generated by natural sources^24,25^. It has been applied in various fields, such as seismic tomography, volcanic activity monitoring, and civil engineering, to identify different types of subsurface structures, volcano hazards, and structural fractures^3,26–28^, allowing to conduct of an effective analysis and monitoring systems. Long-time stacking of ambient noise cross-correlations can extract a high signal-to-noise ratio and stable surface wave signals from the source to the receivers^29^, which are generally stronger and much slower compared with the body waves. Barmin et al.^30^ introduced a method that involves reconstructing the near-field empirical Green’s functions between an arbitrary hypothetical point and remote stations in a closely spaced array with grid search. The approach employs a comparison of the synthetic empirical Green’s functions envelopes with those of earthquake recordings to create an objective function for earthquake location determination. Zhan et al.^31^ introduced a technique that relies on a single nearby station, rather than a densely spaced array in the vicinity of the earthquake, to determine location. This method is calibrated based on the envelope differences between the recorded waveforms from the earthquake and synthetic seismograms generated by assuming a surface load of vertical force at one seismic station. Nevertheless, these methods require a seismic station near the source as a reference event. The grid-search analysis utilizing remote stations verifies that tremor signals can be effectively retrieved from the source, and the envelopes of the empirical Green’s functions may help investigate the wavefield and determine the location of volcanic tremors across large distances^32^. However, the resolution of the energy peak in this waveform-based method generally constrains the potential source location to a relatively large region, and this method is performed by the waveform stacking which requires more computational cost.

**Figure 1:**
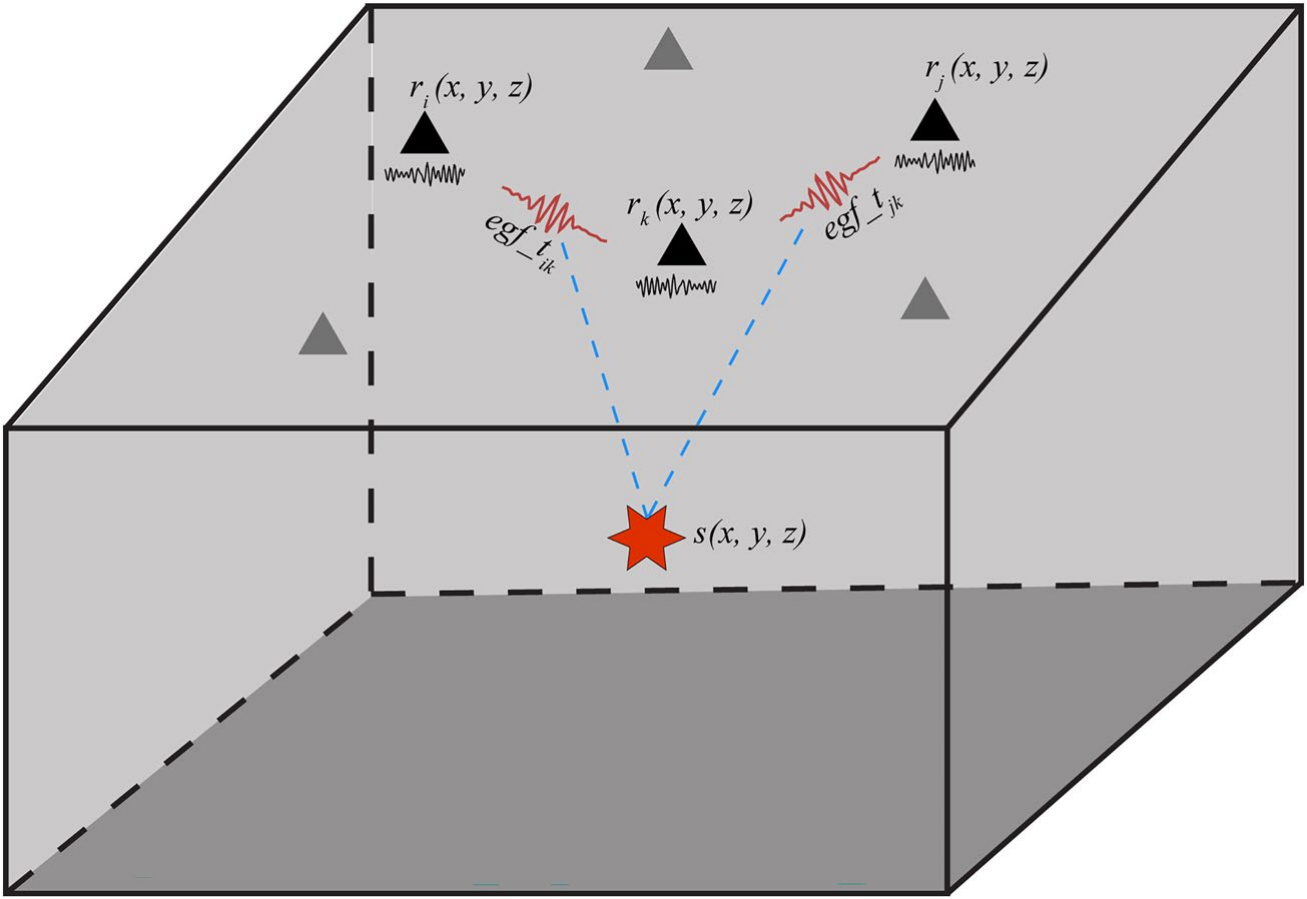
Illustration of regional noise source location based on the station-pair time-delay from ambient noise interferometry. The red star is treated as a potential source. The black triangles are the seismic stations. The black waveforms beneath each station are the raw data and the red waveforms are the empirical Green’s functions derived from ambient noise interferometry between the station-pairs.

In this study, we present a method to detect the regional noise source location based on the station-pair time-delay from ambient noise interferometry in a simplified 3-D homogeneous model (Fig. 1). It is worth noting that the source depth is not well constrained due to the surface waves having little sensitivity for their time-delays in the shallow depth^33^. The time-delays are measured by the cross-correlation waveforms between each station-pair, avoiding phase-picking errors in individual stations. The measured station-pair time-delays are then used to determine the absolute location of the source. This method can accurately and quickly locate the regional source within the framework of ambient noise cross-correlations, which has the capability to detect time-dependent behavior, especially for volcano monitoring.

## Source location based on the station-pair time-delay

The classic source location approach based on the station-pair time-delay has been proposed and applied in the epicentral location of very long period tremor in a 2-D planar location^34,35^, indicating that this method is an efficient technique for locating the source. Here, we exploit the station-pair time-delay source location in a 3-D homogeneous model, considering the surface topography in the process of source detection.

To determine the location of the source *s*(*x*, *y*, *z*), we can use three receivers, receiver *r*_*i*_(*x*, *y*, *z*), receiver *r*_*j*_(*x*, *y*, *z*) and a random reference receiver *r*_*k*_(*x*, *y*, *z*), and *t*_*i*_, *t*_*j*_, and *t*_*k*_ are the arrival times from the source to each receiver, respectively. We assume the velocity (*v*) is uniform in a model space. The distance between the source *s* and the receiver *r*_*i*_ can be calculated as:

1 $$\left\| {s - {r_i}} \right\| = v({t_i} - {t_k}) + \left\| {s - {r_k}} \right\|$$

Taking the squared distance and expanding the equation,

2 $$\left\| {s - {r_i}} \right\|^2 = {v^2}{({t_i} - {t_k})^2} + 2v({t_i} - {t_k})\left\| {s - {r_k}} \right\| + \left\| {s - {r_k}} \right\|^2$$

Then, introducing another receiver *r*_*j*_,

3 $$\left\| {s - {r_j}} \right\|^2 = {v^2}{({t_j} - {t_k})^2} + 2v({t_j} - {t_k})\left\| {s - {r_k}} \right\| + \left\| {s - {r_k}} \right\|^2$$

The term $\left\| {s - {r_k}} \right\|$ can be eliminated,

4 $$v({t_j} - {t_k}) + \frac{{{{\left\| {s - {r_k}} \right\|}^2} - {{\left\| {s - {r_j}} \right\|}^2}}}{{v({t_j} - {t_k})}} = v({t_i} - {t_k}) + \frac{{{{\left\| {s - {r_k}} \right\|}^2} - {{\left\| {s - r} \right\|}_i}^2}}{{v({t_i} - {t_k})}}$$

Expanding this equation, the relationship between the source and three receivers can be described by:

5 $$v({t_j} - {t_k}) + \frac{{2({r_j}^T - {r_k}^T)s + r_k^T{r_k} - r_j^T{r_j}}}{{v({t_j} - {t_k})}} = v({t_i} - {t_k}) + \frac{{2(r_i^T - r_k^T)s + r_k^T{r_k} - r_i^T{r_i}}}{{v({t_i} - {t_k})}}$$

Note that, the inversion problem becomes solving for *s* in

6 $$Gs = d,$$

where

$$G = 2(v({t_j} - {t_k})({r_i} - {r_k}) - v({t_i} - {t_k})({r_j} - {r_k}))$$

$$d = v({t_i} - {t_k})({v^2}{({t_j} - {t_k})^2} - {r_j}^T{r_j}) + (v({t_i} - {t_k}) - v({t_j} - {t_k})){r_k}^T{r_k} + v({t_j} - {t_k})({r_i}^T{r_i} - {v^2}{({t_i} - {t_k})^2})$$

The model parameter *s* is the location of the source. This problem can be solved using a standard least squares scheme (Text 1 in the Supplementary).

## Synthetic tests

**Figure 2:**
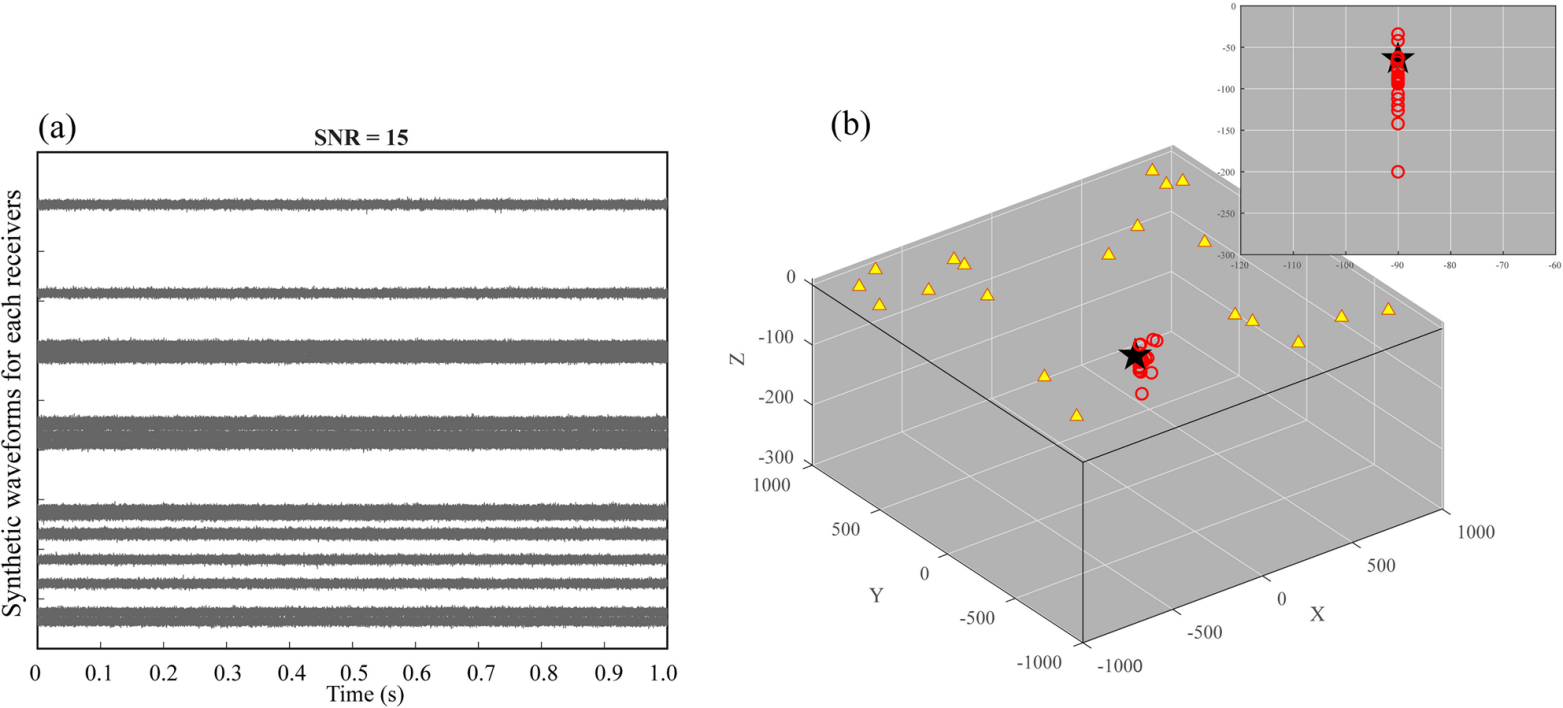
The accuracy of the estimated source location using synthetic waveforms with signal-to-noise ratio of 15. (**a**) Random synthetic waveforms are generated with SNR = 15. (**b**) Bootstrap sampling analyses for evaluating the uncertainty of the source location. The yellow triangles represent the receivers and the black star represents the location of the source. The red circles represent the estimated source locations. The inset shows the estimated source locations as viewed from the YZ plane.

We performed synthetic tests to validate the method in a 3-D homogeneous model. The horizontal ranges (X and Y axes) vary between − 1000 and 1000 m, and the vertical ranges (Z axis) vary between − 300 and 5 m (Fig. 2). The particular type of seismic source is generated by the ${\text{sinc}}(100\pi {\text{x}})$ function that is generally used in geophysical signal processing^36^. In this test, the spatial grid has been defined with a uniform velocity of 1500 m/s, and the random 20 receivers are produced by this synthetic source in terms of random locations of the receivers. All receivers have been positioned at a depth range of 0–5 km due to the true station position being situated above the surface. The synthetic waveforms are generated by shifting the seismic source signal according to the distance between the source and the receivers. We added a Gaussian distribution noise with varying levels of amplitude to the synthetic waveform. Here, the signal-to-noise ratio is defined as $SNR = 10\;log\;({S^2}{/}{N^2})$, where *S* represents the maximum amplitude of the signal and *N* represents the root mean square of the amplitude in the noise^37^. The time-delay measurements are taken from the average value of the cross-correlation in the causal or acausal part between each receiver pair. The cross-correlation is defined in the time domain and is computed within localized time windows, allowing for an estimation of the time-dependent similarity between two synthetic waveforms (Fig. S2).

**Figure 3:**
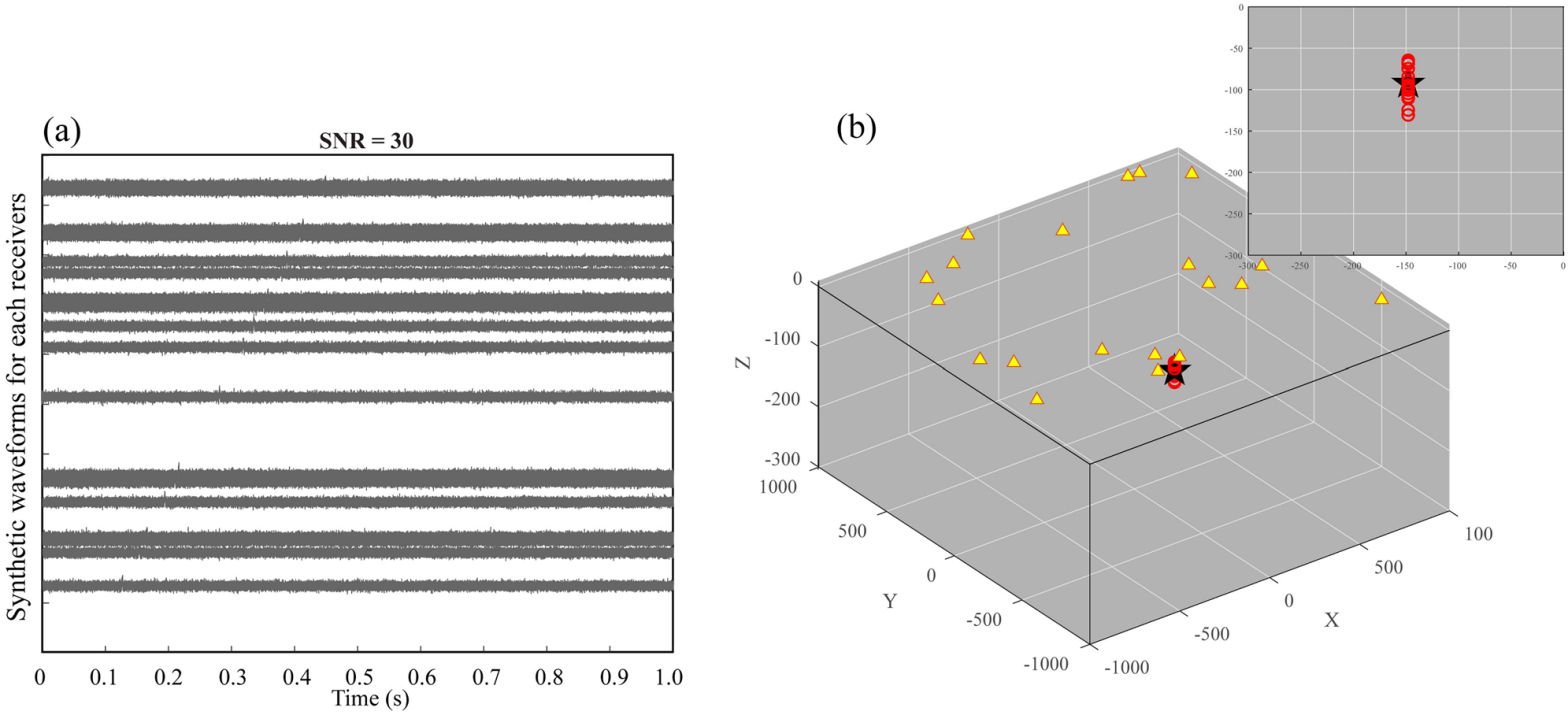
The same as Fig. 2 but with signal-to-noise ratio of 30. The estimated source locations in (**b**) are close to the seismic source. See the descriptions in Fig. 2 for other labels.

**Figure 4:**
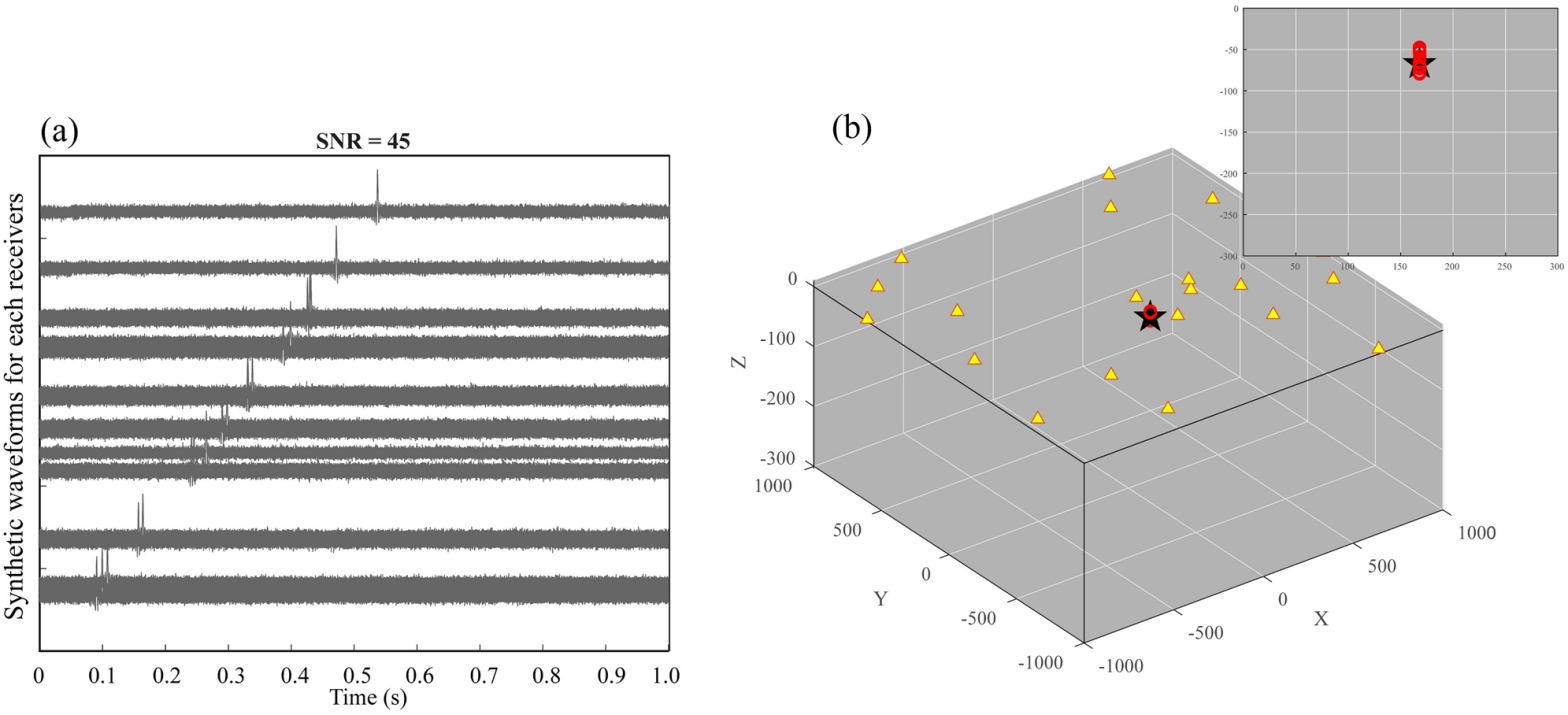
The same as Fig. 2 but with signal-to-noise ratio of 45. The estimated source locations in (**b**) are concentrated on the seismic source. See the descriptions in Fig. 2 for other labels.

**Figure 5:**
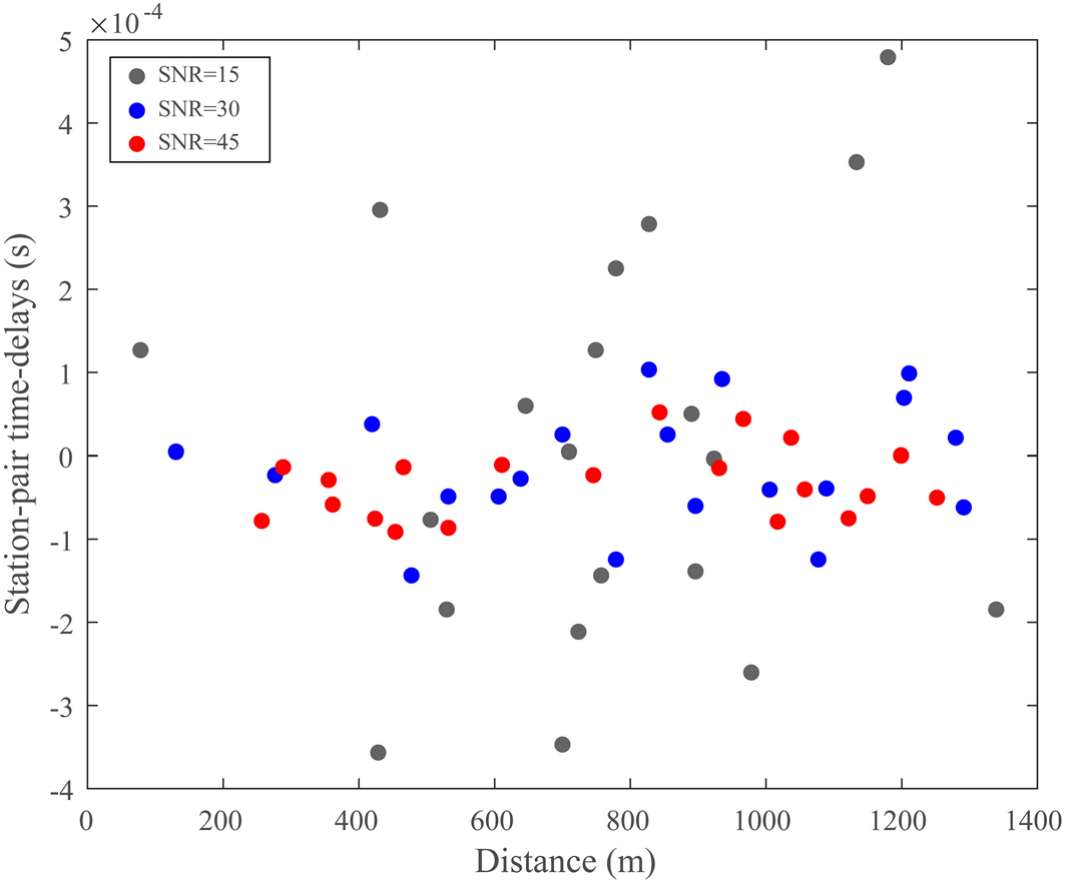
The distribution of the differences between the theoretical traveltimes and the observed traveltimes obtained from cross-correlations for different signal-to-noise ratio values at different distances from the same source to receivers.

The signal-to-noise ratio of the waveform is the significant factor for the detection and location of the source. Here we test the impact of the signal-to-noise ratio on the uncertainty of the source location. We define three different signal-to-noise ratio synthetic waveforms for the uncertainty assessment (Figs. 2, 3, 4), which help in understanding the impact of noise on the source location. In the case of a relatively low signal-to-noise ratio (SNR = 15), the seismic source is randomly located at *x* = − 24 m, *y* = − 90 m, and *z* = − 65 m. As shown in Fig. 2a, the seismic source signals are embedded within the random noise for each synthetic waveform, and the amplitude of the signal is approximately equal to that of the noise. In this case, the identification of the source therefore has a large error because of slight distinctions between the random noise and the source. We performed 20 bootstrap sampling analyses to evaluate the uncertainty of the source location. The bootstrap samples are generated by randomly sampling from the time-delays between the receivers, and we estimate the source location based on each bootstrap sample. Then, we statistically analyze the accuracy of the estimated source location. The estimated locations are generally distributed in a relatively small region surrounding the actual seismic source (Fig. 2b). The standard deviation of the difference is 28.08 m. In addition, the other two tests are carried out to verify the impact of the signal-to-noise ratio on the uncertainty of the source location. In the case of SNR = 30, the seismic source is randomly located at *x* = 113 m, *y* = − 148 m, and *z* = − 94 m. As shown in Fig. 3a, the amplitude of the signal at each receiver can be barely recognized. The approximated positions tend to cluster closely to the origin of the seismic source (Fig. 3b), and the standard deviation of the difference is 5.42 m. In the case of SNR = 45, the random seismic source is located at *x* = 249 m, *y* = − 168 m, and *z* = − 67 m. The signals can be obviously distinguished from the noise (Fig. 4a). Thus, the estimated locations are most concentrated on the seismic source (Fig. 4b), in correspondence with the minimum standard deviation (1.61 m). We calculate the differences between the theoretical traveltimes and the observed traveltimes obtained from cross-correlations for different SNR values at different distances with the same source and receivers. As shown in Fig. 5, the differences are obviously larger for the SNR = 15 case in comparison with the other cases, indicating that the SNR of the synthetic waveform plays a crucial role in detecting and locating the source.

**Figure 6:**
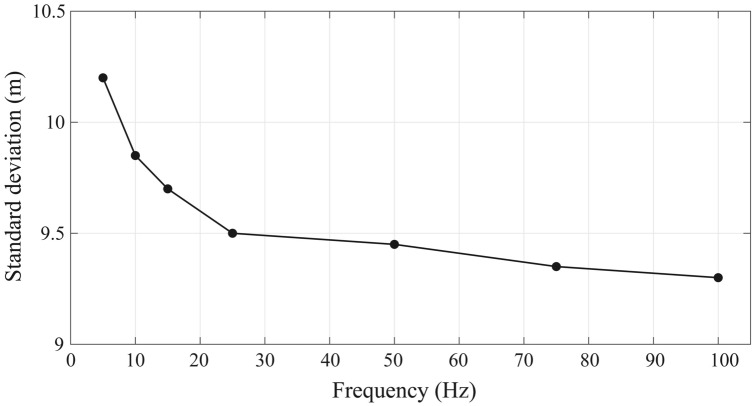
The standard deviation of the difference between the estimated source and real source in different frequencies.

Subsequently, we test how the frequency bandwidth of the source influences the accuracy of calculating the time-delay using the cross-correlations. The synthetic waveforms are produced by seven different frequency bandwidths, ranging from 5 Hz up to 100 Hz, with the same signal-to-noise ratio of 20. Figure 6 shows the standard deviation of the difference between the estimated source and the real source in different frequencies. The large standard deviations are observed in the relatively lower frequencies (< 15 Hz), and there is a noticeable trend of decreasing standard deviations with increasing frequency. According to the results, it can be concluded that the accuracy is closely related to the dominant wavelength of the source. Within the resolution of band-limited signals, the higher frequency bandwidth source signals are more accurate in detecting and locating sources. We can robustly retrieve the location of the source in terms of the dominant wavelength to fulfill different monitoring purposes. Furthermore, the estimated source location is also influenced by the azimuth angle of the receiver locations. In our synthetic tests, the locations of the receivers are randomly generated, however, the uneven distributions of the locations do not severely affect the accuracy of the source location (Figs. 3 and 4). Actually, the variations in receiver distributions can lead to errors in source location that cannot be quantified directly.

## Application

**Figure 7:**
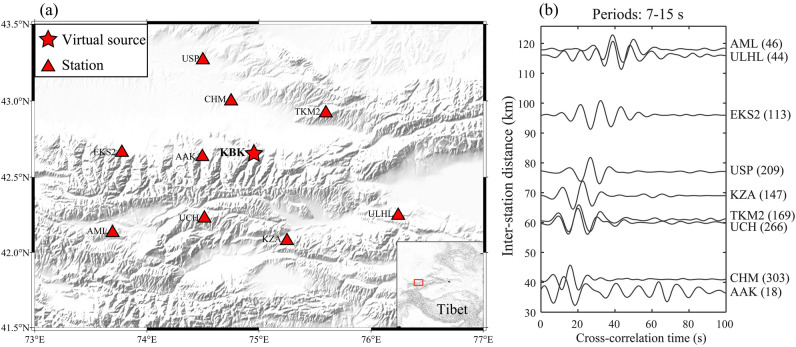
(**a**) The distribution of seismic stations from the Kyrgyz Seismic Telemetry Network in the Central Tien Shan. The KBK station (red star) is designated as a virtual source. (**b**) The empirical Green’s functions between each station-pair. The number on the right of each station-pair name represents the signal-to-noise ratio value. Imagery is available from the U.S. Geological Survey (https://lpdaac.usgs.gov/products/srtmgl1v003). Figure made with Generic Mapping Tools^39^ (GMT v.6.4.0: https://www.generic-mapping-tools.org).

Typically, assessments of the noise source location accuracy rely on ground truth locations. However, the precise location of the source is unknowable. To mitigate this issue, we present a test that treats the seismic station as a virtual source for determining its location. We consider the Kyrgyz Seismic Telemetry Network (KN) which consists of 10 broadband stations in the Central Tien Shan (Fig. 7a). The KBK station is designated as a virtual source at the center of the KN array, and we then attempt to determine the location of this station by analyzing the empirical Green’s functions between all of the other stations with the KBK station. The ambient noise interferometry is dominantly composed of surface waves at periods larger than 5 s in the study region^26,38^. Receiver stations located in the vicinity of the virtual source tend to retain more pronounced amplitudes due to surface waves are most sensitive to the near-surface structures in the shorter distance, which allows for more accurate detection and measurement of the surface waves, and can help in determining the noise source location with low uncertainty. The virtual source is not considered as one of the receivers during the inversion process. Another advantage of this approach is that it eliminates the influence of unknown source mechanisms and depth on the determination of the location.

The Rayleigh wave empirical Green’s functions are derived by analyzing the cross-correlation of ambient noise waveforms between pairs of stations during 1997–2000, following the method described by Lü et al.^26^. To ensure reliable empirical Green’s functions and enhance the signal-to-noise ratio, we first remove the instrument response and then truncate the resulting displacement seismogram to a daily duration. The daily data is resampled to a sample rate of 10 Hz. Generally, the low frequency surface waves are often dominant in the cross-correlation, however, the high frequency body waves are also observed and likely entangled with the surface waves in previous studies^3,17^. The ambient noise data are normalized with the frequency-time normalization^40^. Each waveform is filtered within a frequency band of surface wave (0.01–0.4 Hz) and then is divided by its corresponding envelope to create a time series with unit amplitude. We also stack the cross-correlations to suppress body-wave content and enhance the visibility of surface waves. For more details, refer to the Text 2 in the Supplementary. As demonstrated in Fig. 7b, we are able to extract high-quality Rayleigh wave empirical Green’s functions at periods ranging from 7 to 15 s with a relatively high signal-to-noise ratio.

**Figure 8:**
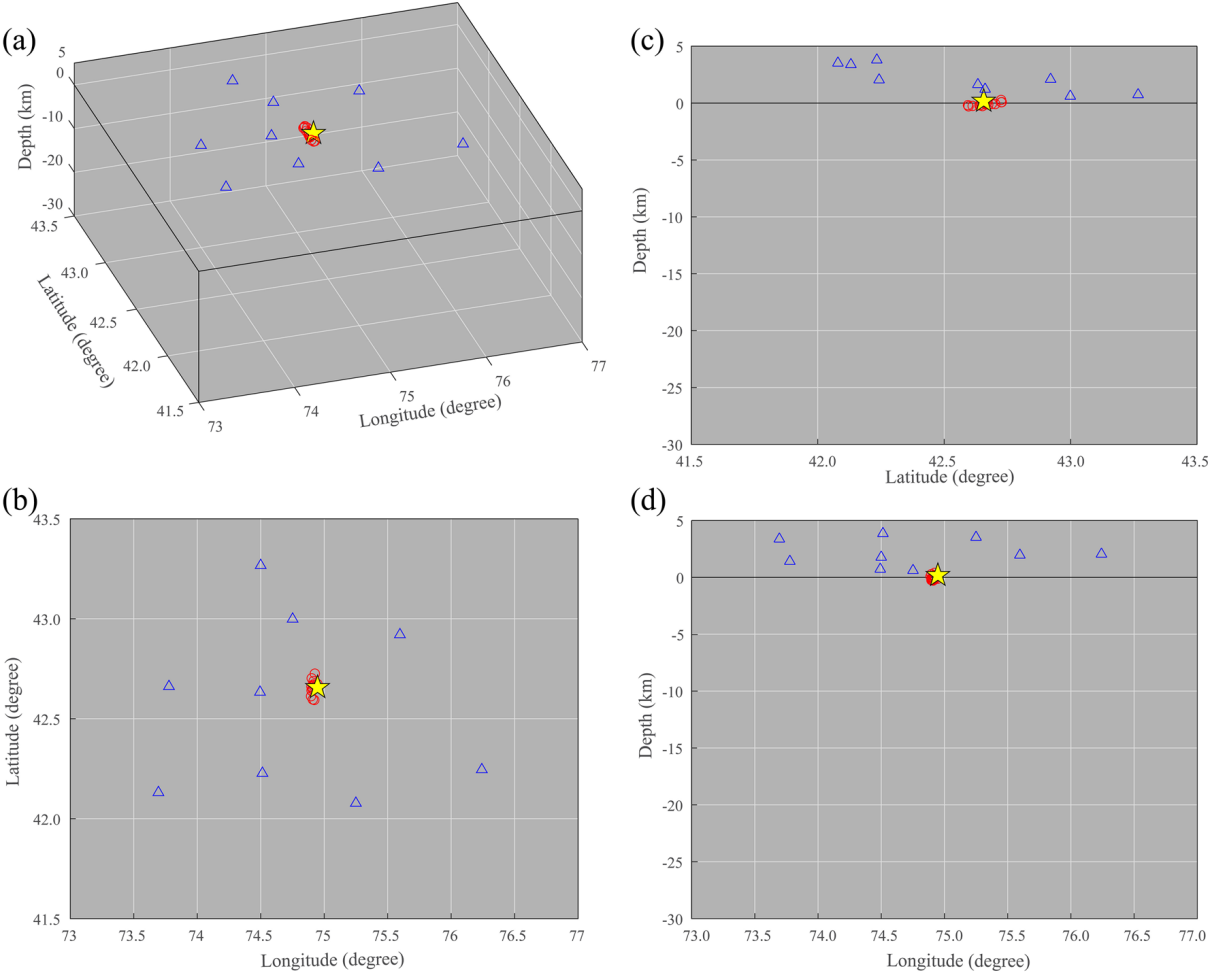
The results of virtual source location in different views. The blue triangles represent the seismic stations and the yellow star represents the location of the virtual source. The red circles represent the estimated source locations.

We choose the empirical Green’s functions derived from KBK-CHM station-pair that has the highest signal-to-noise ratio as a reference waveform. The time-delays between each station-pair are measured using cross-correlation calculations to determine the absolute location of the source (known as the KBK location.). The constant propagation velocity of 3.078 km/s is used, which is determined by performing a linear least square regression on the empirical Green’s functions at periods ranging from 7–15 s within the signal time window. The average interstation spacing of seismic stations in the study area is ~ 80 km. The results of the virtual source location test are presented in Fig. 8. We also generate 20 bootstrap samples by randomly sampling from the station-pair time-delays and estimate the virtual source location based on each bootstrap sample. Then, we statistically analyze the accuracy of the virtual source location. The spatial distributions of the locations are basically concentrated on the hypocenter location of the virtual source. The location errors are 3.64 km and 3.25 km in the horizontal direction and 0.14 km in depth. The corresponding standard deviations are 0.0409, 0.0125, and 0.0197 km, respectively. These results demonstrate that the proposed algorithm can achieve the potential source location based on the station-pair time-delay from ambient noise interferometry for the regional station coverage.

## Discussion and conclusion

We introduce a source location method based on the station-pair time-delay from ambient noise interferometry. This method implements the estimation of the source location by utilizing a set of station-pair time-delay and assuming uniform propagation velocity. We tested this method with a set of synthetic signals and illustrated it with an application to the real data records from 10 broad-band seismic stations installed in the Central Tien Shan. Our method can accurately locate the noise source based on ambient noise interferometry in specific periods rather than projecting the energy of the source onto multiple grid points^1,32,41^, and the total computing time in our application only requires a few seconds. In addition, the back projection method typically limits the potential source location to a specific region, whereas we can determine the absolute source location based on the observed time-delays between station-pairs. In comparison with the reference station method, a fixed master station is selected by referring to the source, our method avoids the selection of the master station to reduce the artificial factors affecting the accuracy of the source location.

In the synthetic tests, we are able to determine the source location with a high accuracy despite the presence of different types of added noise in the seismograms. The signal-to-noise ratio of the synthetic waveform is a significant factor in locating the source. A higher SNR generally results in a more accurate and precise estimation of the source location due to the strong signal in comparison to the noise makes it easier to distinguish the true signal from the noise, leading to a more reliable localization of the source. On the other hand, a lower SNR can significantly increase the uncertainty in the estimated source location. It becomes challenging to accurately determine the source location in situations where the signal is weak compared to the noise. In general, unless the added noises have amplitudes comparable to that of the source, they will not have a significant effect on its source-location estimation in the synthetic tests. Furthermore, the frequency bandwidth of the source is an additional consideration for detecting the signals during the cross-correlation process. Based on the conducted tests, it can be observed that the precision of source localization is somewhat influenced by the frequency. This result implies that a higher frequency generally leads to a more precise estimation of the source location.

We then applied our method to the data from the Kyrgyz Seismic Telemetry Network at the Central Tien Shan. Our method can determine the assumed virtual source location based on the station-pair time-delay from ambient noise interferometry with an accuracy within a few kilometers. The ambient noise interferometry is dominantly composed of surface waves at periods larger than 5 s in the Central Tien Shan^26,38^. Due to the intrinsic limitation of surface waves, it is difficult to ascertain the depth of the seismic source. As shown in Fig. S1, the frequency-dependent surface wave in fundamental mode has a broader depth sensitivity kernel. For example, the 10 s period surface wave is more sensitive to the depths of 7–15 km, whereas the 15 s period surface wave is more sensitive to the depths of 11–22 km. Moreover, the amplitudes of sensitivity are also reduced as the period increases. It is challenging to accurately estimate the depth of a source solely relying on surface waves. Although our method makes several simplifications concerning a 3-D homogeneous model, the depth of the source can be generally recovered from ambient noise interferometry. Ambient noise interferometry provides continuous and long-term information on the subsurface structure without the need for specific earthquakes, which is suitable for detecting time-dependent behavior, such as industrial monitoring and volcano monitoring. There are remaining questions to be addressed by future studies. The integration of denser station coverage with artificial intelligence and machine learning would further improve the precision of noise sources.

In this study, we have not addressed all potential complexities and challenges associated with source location from ambient noise interferometry, including multiple dominant noise sources and complex station geometries. Such problems may be mitigated by further processing techniques. The presented method here provides a new scheme to determine the absolute location of the source based on the station-pair time-delay from the ambient noise interferometry without performing waveform stacking. By integrating the advantage of ambient noise interferometry, we have the capability to monitor the time-varying behavior of the source and enhance our understanding of the dynamic processes associated with complex source activities.

### Supplementary Information

Supplementary Information.

## Data Availability

The data underlying this article are available in the IRIS Data Management Center at https://ds.iris.edu/ds/nodes/dmc/.
