# Supplementary material for: Regional noise source location based on the time delays between station pairs from ambient noise interferometry
Source: Sci Rep. 2024 May 4;14:10268. doi: 10.1038/s41598-024-60313-1 (PMC11069535; doi:10.1038/s41598-024-60313-1)
Supplement: Supplementary file 1 — Supplementary Information. [file 41598_2024_60313_MOESM1_ESM.pdf]

Supplementary Materials

Regional noise source location based on the station-pair time-delay from ambient noise interferometry

Ziqiang Lü[1], Qian Liu[1], Qinghan Kong[1], Jingwen Sun[1], Zemin Liu[2]

[1]*College of Mining, Liaoning Technical University, 123000 Fuxin, China*

[2]*Institute of Geophysics, China Earthquake Administration, 100081 Beijing, China*

**Corresponding authors: Ziqiang Lü (ziqianglyu@sina.com)*

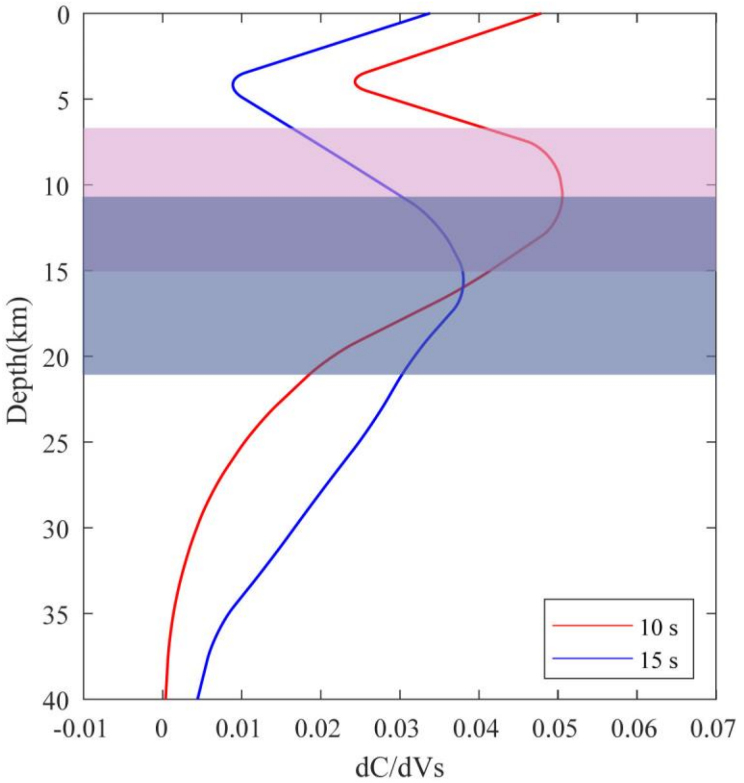

Supplemental Figure 1. Depth sensitivity kernels of Rayleigh-wave phase velocity in fundamental mode at 10 s and 15 s periods.

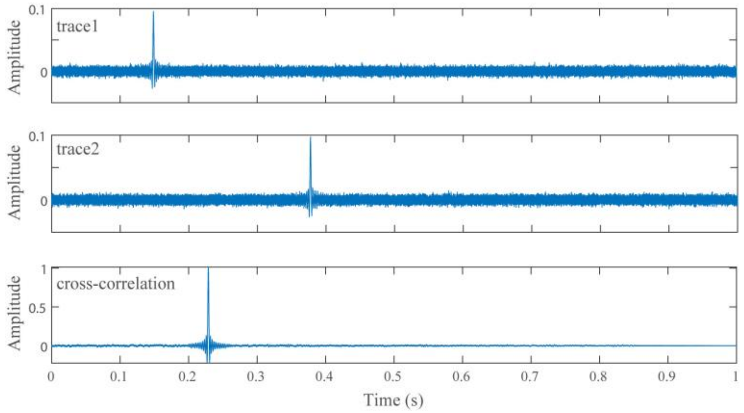

Supplemental Figure 2. Example of cross-correlated results between two synthetic waveforms

Supplementary Text 1.

In the inversion processes, the least squares scheme is used to estimate the parameters of the source location. The relationship between the source and receivers can be described by:

$$Gs = d$$

where

$G=2(v(t_j\text{-}t_k)(r_i\text{-}r_k)\text{-}v(t_i\text{-}t_k)(r_j\text{-}r_k))$

$d=v(t_i\text{-}t_k)(v^2(t_j\text{-}t_k)^2\text{-}r_j^T r_j)+(v(t_i\text{-}t_k)\text{-}v(t_j\text{-}t_k))r_k^T r_k+v(t_j\text{-}t_k)(r_i^T r_i\text{-}v^2(t_i\text{-}t_k)^2)$

The least-squares solution of $Gs = d$ is the solution of the matrix equation

$$G^T Gs = G^T d$$

In the following case, $Gs = d$ has a unique least-squares solution, the columns of G are linearly independent, and $G^T G$ is invertible. The least-squares solution is

$$s = (G^T G)^{-1} G^T d$$

For multiple receivers, we can create a bigger matrix of the time-delays between the receivers for solving the location of the source.

Supplementary Text 2.

The Rayleigh wave empirical Green’s functions (EGFs) are extracted from the vertical-component ambient noise cross-correlation of the waveforms between each station pair. Prior to cross-correlating the waveforms between station pairs, we remove the instrument response, normalize the ambient noise data with a frequency-time

normalization method (Shen et al., 2012), and eliminate waveform segments for large earthquakes (magnitude ⩾5.5). The frequency-time normalization method constructs seismograms with an even spectrum at all times within the data processing unit. Specifically, it filters the original seismogram using a series of narrow-frequency band-pass filters within the range of 0.01-0.4 Hz. Then it divides each filtered seismogram by its envelope, computed as the Hilbert transform of the seismogram. The filtered and normalized seismograms are summed for all frequency bands to produce the final frequency-time normalized seismogram. Benefiting from the frequency-time normalization method, we are able to extract high-quality Rayleigh wave signals.
